# The UCEIS and UC-100 score were responsive endoscopic and global indices in a phase 2 trial of ulcerative colitis

**DOI:** 10.1093/crocol/otag031

**Published:** 2026-04-24

**Authors:** Sudheer K Vuyyuru, Simon Travis, Soushyant Kiarasi, Guangyong Zou, Stephen Lagana, David F Schaeffer, Vipin Arora, Jay L Tuttle, Christopher Ma, Vipul Jairath

**Affiliations:** Alimentiv Inc., London, ON, Canada; Division of Gastroenterology, Schulich School of Medicine & Dentistry, Western University, London, ON, Canada; Kennedy Institute and Biomedical Research Centre, University of Oxford, Oxford, United Kingdom; Alimentiv Inc., London, ON, Canada; Alimentiv Inc., London, ON, Canada; Department of Epidemiology & Biostatistics, Western University, London, ON, Canada; Robarts Research Institute, Schulich School of Medicine and Dentistry, University of Western Ontario, London, ON, Canada; Department of Pathology and Cell Biology, Columbia University Medical Center, New York City, NY, United States; Division of Anatomic Pathology, Vancouver General Hospital, Vancouver, BC, Canada; Eli Lilly and Co., San Diego, CA, United States; Eli Lilly and Co., San Diego, CA, United States; Alimentiv Inc., London, ON, Canada; Division of Gastroenterology & Hepatology, University of Calgary, Calgary, AB, Canada; Department of Community Health Sciences, University of Calgary, Calgary, AB, Canada; Alimentiv Inc., London, ON, Canada; Division of Gastroenterology, Schulich School of Medicine & Dentistry, Western University, London, ON, Canada; Department of Epidemiology & Biostatistics, Western University, London, ON, Canada

**Keywords:** evaluative instruments, endoscopy, responsiveness

## Abstract

**Background:**

Trials evaluating ulcerative colitis (UC) therapies necessitate instruments that are responsive to changes in disease status. We assessed responsiveness of the Mayo Endoscopic Subscore (MES), Ulcerative Colitis Endoscopic Index of Severity (UCEIS), Robarts Histopathology Index (RHI), Geboes Score (GS), Nancy Histological Index (NHI), and UC-100 score.

**Methods:**

Using data from a phase 2 placebo-controlled trial of mirikizumab, responsiveness was quantified by the probability that a patient in the treatment group would have a better score than (win over) a control patient. Inference on win probability (WinP) was conducted without normality assumption of scores. Spearman rank correlations between each index and stool frequency (SF), rectal bleeding (RB), fecal calprotectin (FCAL), and C-reactive protein (CRP) were assessed.

**Results:**

WinP estimates showed that magnitudes of responsiveness were “medium” for the UCEIS (0.67; 95% CI 0.59, 0.74) and UC-100 score (0.68; 95% CI 0.60, 0.75) and “small” for the RHI (0.62; 95% CI 0.54, 0.70), MES (0.61; 95% CI 0.53, 0.68), and GS (0.60; 95% CI 0.52, 0.68). The NHI showed no statistical evidence of responsiveness (0.57; 95% CI 0.48, 0.65). The UCEIS and MES had weak-to-moderate correlations with SF (0.21-0.26) and RB (0.29-0.33) but no correlation with biomarkers. The UC-100 had moderate correlations with RB (0.46) and FCAL (0.42) and weak correlation with CRP (0.19).

**Conclusions:**

The UCEIS and UC-100 score were responsive to changes in endoscopic and global UC disease activity, respectively. Results may help inform future early phase UC drug trials but should be confirmed in larger studies.

## Graphical Abstract

**Graphical Abstract:**
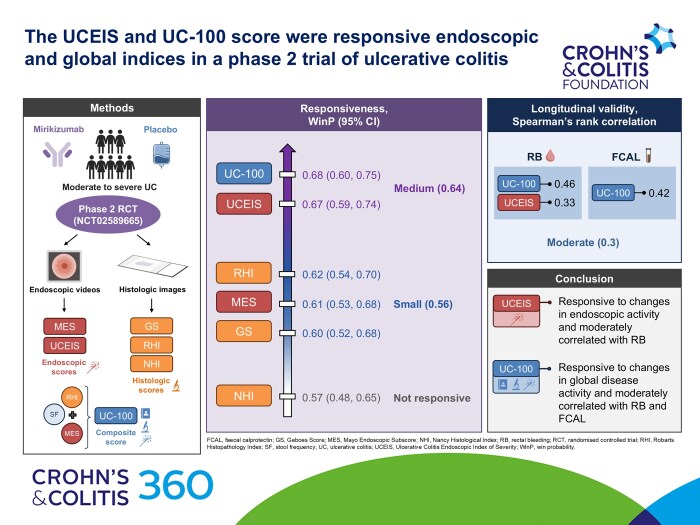

## Introduction

Ulcerative colitis (UC) is a chronic inflammatory bowel disease with a variable clinical course. Assessment of disease activity and response to therapy in patients with UC is typically measured by clinical, endoscopic, histologic, and biochemical examination. Achievement of endoscopic healing is associated with a higher likelihood of corticosteroid-free remission and a decreased risk of relapse and colectomy.^1^ Consequently, regulatory authorities have mandated the inclusion of endoscopic endpoints in clinical trials.^2^ The Selecting Therapeutic Targets in Inflammatory Bowel Disease (STRIDE)-II guidelines recommend achievement of endoscopic remission as a long-term treatment goal in UC, with histologic remission positioned as an adjunctive target.^3^

The Mayo Clinic Score (MCS) was developed in 1987 and consists of four variables: stool frequency (SF), rectal bleeding (RB), a physician’s global assessment (PGA), and endoscopic findings with flexible sigmoidoscopy (the Mayo Endoscopic Subscore [MES]).^4^ The MES assesses endoscopic activity on a 4-point scale (0-3 points), with higher scores representing more severe disease. Draft guidance issued by the U.S. Food and Drug Administration in 2016 introduced several modifications to the MCS such as exclusion of the PGA from the total score and removal of mild friability from the definition of a MES of 1. The resulting three-component modified MCS has been accepted as a standard endpoint in UC clinical trials.^2^

In 2012, Travis and colleagues developed the Ulcerative Colitis Endoscopic Index of Severity (UCEIS) after identifying three descriptors (vascular pattern, bleeding, and erosions and ulcers) that were most predictive of overall UC disease severity, as measured by a visual analogue scale (VAS).^5^ Scoring of each descriptor is performed on a 3- or 4-point Likert scale, with total scores ranging from 0 to 8.^6^

The UCEIS has been shown to correlate closely with validated histologic indices, including the Robarts Histopathology Index (RHI) and Nancy Histological Index (NHI).^7^ Histopathology has prognostic value in UC, especially when there is concordance between remission and endoscopy.^8^^,^^9^ Prospective correlation between symptoms, the UCEIS, the NHI, and fecal calprotectin (FCAL) has also been performed, providing novel FCAL thresholds for defining endoscopic and histologic disease activity.^10^ Furthermore, emerging data from several drug development programs in UC suggest that histology may be a relevant endpoint for induction and maintenance trials.^11^^,^^12^ Selecting the most responsive evaluative instrument is especially important in early drug development stages to inform go/no-go decisions and can influence later-stage trial design. The aims of this study were to assess and compare the responsiveness of existing endoscopic (MES, UCEIS), histologic (RHI, Geboes Score [GS], NHI), and composite (UC-100) indices and to evaluate the correlation between the change in these indices and the change in clinical and biomarker outcomes.

## Methods

### Study population and design

This post hoc analysis used existing clinical trial data from a phase 2, randomized, double-blind, parallel, placebo-controlled study of mirikizumab in patients with moderately to severely active UC (NCT02589665), in which 249 patients were assigned to receive intravenous placebo (*n* = 63), mirikizumab 50 mg with exposure-based dosing (*n* = 63), mirikizumab 200 mg (*n* = 62) with exposure-based dosing, or mirikizumab 600 mg with fixed dosing (*n* = 61) at weeks 0, 4, and 8.^13^ The primary endpoint was the proportion of patients in clinical remission at week 12, defined as a Mayo RB subscore of 0, a SF subscore of 0 or 1 with at least a 1-point decrease from baseline, and an MES of 0 or 1. Video-recorded endoscopy with biopsy collection was performed at baseline, week 12, and week 52. Endoscopic findings at baseline and each efficacy assessment were scored by a central reader using the MES and UCEIS. Histologic disease activity was evaluated at baseline and each efficacy assessment using the GS. This evaluation was conducted by a central reader who analyzed samples from two biopsies obtained from the most severely affected area, located at least 30 cm from the anal verge.^13^

Re-reading of endoscopy videos was not performed in the current study; MES and UCEIS data obtained in the original trial were utilized. Likewise, histologic evaluations employing the GS from the original trial were incorporated. RHI scores were subsequently derived from existing GS data, and UC-100 scores were calculated. The UC-100 is a composite index that incorporates three components (calculated as 1 + [16 × Mayo SF subscore] + [6 × MES] + [1 × RHI]) and ranges from 1 (no disease activity) to 100 (severe disease activity).^14^ For the purposes of this study, a blinded central reader scored all available baseline and week 12 histologic image pairs to generate NHI scores. Image pairs were only assessed if the central reader rated both samples as being of adequate quality. Data from the mirikizumab 50 mg group were excluded from all analyses due to lower efficacy, and no post hoc efficacy or safety analyses of NCT02589665 were conducted.

### Study objectives

The primary study objectives were to (1) assess and compare the responsiveness of endoscopic indices (MES, UCEIS), histopathological indices (GS, RHI, NHI), and a composite index (UC-100) (Supplementary Table S1)^4^^,^^5^^,^^14–17^; and (2) evaluate the correlation between change in endoscopic, histologic, and composite indices with change in clinical (SF, RB loss), and biomarker (FCAL and C-reactive protein [CRP]) outcomes in patients who received mirikizumab, a treatment of known efficacy, or placebo. For the primary analysis, the mirikizumab 200 mg and 600 mg arms were pooled into a single treatment group.

### Statistical analysis

Descriptive statistics were used to characterize the baseline demographics of patients included in the study.

Responsiveness was assessed using an effective treatment (mirikizumab) as the anchor. The magnitude of responsiveness was quantified as the probability that a patient in the treatment group would have a better disease activity score than (or win over) a control patient (placebo). This approach to responsiveness assessment is particularly useful for disease scores that lack meaningful units. Specifically, this win probability (WinP) directly answers the following question: “What is the probability that a treated participant has a more favorable outcome than a control participant?” It has been noted that WinP is the parameter underlying the Mann-Whitney nonparametric test.^18^ The WinP has a dozen different names in the literature,^19^ including the area under the receiver operating characteristic curve. When data are normally distributed with common variance in two comparison groups, the WinP can be directly converted to the standardized mean difference (Cohen’s effect size) (i.e., $\text{effect}\;\text{size}=\sqrt{2}\times \text{inverse}\;\text{normal}\;[\text{WinP}]$). This relationship suggests that WinP estimates of 0.50, 0.56, 0.64, and 0.71 correspond to Cohen’s effect sizes of 0, 0.2, 0.5, and 0.8, respectively, which are commonly interpreted as “null,” “small,” “medium,” and “large” effect sizes.^19^ Comparisons of WinP estimates between different instruments were conducted using the DeLong method as described by Zou et al.^20^

Longitudinal validity was assessed with correlations between the change in the endoscopic, histologic, and composite indices and changes in clinical and biomarker outcomes. Potential violation of normality was overcome by using Spearman’s rank correlation coefficients and associated two-sided 95% CIs by Fisher’s transformation. Correlations were interpreted using Cohen’s benchmarks (coefficients of 0.1, 0.3, and 0.5 represented “weak,” “moderate,” and “high” correlations, respectively).^21^ All analyses were conducted using SAS version 9.4.5.0 (SAS Institute Inc.; Cary, North Carolina, USA).

### Ethical considerations

NCT02589665 was registered with ClinicalTrials.gov, conducted in compliance with the Declaration of Helsinki and International Conference on Harmonisation Good Clinical Practice Guidelines, and approved by the Institutional Review Boards and/or Independent Ethics Committees at each investigational center participating in the study or at a central Institutional Review Board. All participants provided written informed consent specifying that collected data could be used for future research purposes.

## Results

### Baseline characteristics

A total of 177 participants were included in the final analysis after excluding the mirikizumab 50 mg arm (*n* = 63) and patients without paired baseline and week 12 histologic images (*n* = 9). Baseline characteristics of the study participants are presented in Table 1.

**Table 1:** Baseline characteristics.

Parameter	Placebo (*n* = 60)	Mirikizumab 200 mg (*n* = 60)	Mirikizumab 600 mg (*n* = 57)	Pooled (*n* = 117)
**Sex**				
** Female**	27 (45.0%)	24 (40.0%)	20 (35.1%)	44 (37.6%)
** Male**	33 (55.0%)	36 (60.0%)	37 (64.9%)	73 (62.4%)
**Smoking status**				
** Current**	2 (3.3%)	4 (6.7%)	2 (3.5%)	6 (5.1%)
** Former**	15 (25.0%)	21 (35.0%)	18 (31.6%)	39 (33.3%)
** Never**	43 (71.7%)	35 (58.3%)	37 (64.9%)	72 (61.5%)
**Disease location and extent**				
** Left-sided colitis (to splenic flexure)**	21 (35.0%)	30 (50.0%)	21 (36.8%)	51 (43.6%)
** Other**	0 (0.0 %)	1 (1.7%)	1 (1.8%)	2 (1.7%)
** Pancolitis**	10 (16.7%)	13 (21.7%)	14 (24.6%)	27 (23.1%)
** Portion of transverse colon**	2 (3.3%)	5 (8.3%)	4 (7.0%)	9 (7.7%)
** Proctitis (< 15 cm)**	1 (1.7%)	0 (0.0 %)	0 (0.0 %)	0 (0.0 %)
** Proctosigmoiditis (15-25 cm from anal verge)**	26 (43.3%)	11 (18.3%)	17 (29.8%)	28 (23.9%)
**Exposure to biologics**				
** No**	22 (36.7%)	20 (33.3%)	21 (36.8%)	41 (35.0%)
** Yes**	38 (63.3%)	40 (66.7%)	36 (63.2%)	76 (65.0%)
**Age (y)**	*n* = 60	*n* = 60	*n* = 57	*n* = 117
** Mean (SD)**	50.02 (13.4)	50.57 (14.6)	49.70 (13.4)	50.15 (14.0)
**Months since first diagnosis**	*n* = 60	*n* = 60	*n* = 57	*n* = 117
**Median (min, max)**	71.5 (3.0, 443.0)	73.0 (10.0, 587.0)	54.0 (2.0, 265.0)	69.0 (2.0, 587.0)
**CRP (mg/L)**	*n* = 60	*n* = 60	*n* = 57	*n* = 117
**Mean (SD)**	8.8 (13.6)	9.2 (13.0)	14.2 (26.3)	11.6 (20.6)
**Median (min, max)**	4.1 (0.1, 85.6)	3.6 (0.1, 79.1)	6.1 (0.1, 164.0)	4.3 (0.1, 164.0)
**FCAL (mg/kg)**	*n* = 30	*n* = 29	*n* = 26	*n* = 55
**Mean (SD)**	1990.4 (2067.2)	2146.2 (2924.7)	2256.0 (2541.3)	2198.1 (2725.5)
**Median (min, max)**	1157.5 (134.0, 8696.0)	1630.0 (15.0, 16135.0)	1585.0 (106.0, 11353.0)	1630.0 (15.0, 16135.0)
**RHI**	*n* = 59	*n* = 60	*n* = 56	*n* = 116
**Mean (SD)**	17.8 (7.7)	15.5 (9.4)	17.3 (8.7)	16.4 (9.1)
**Median (min, max)**	17.00 (0.0, 33.0)	16.00 (0.0, 33.0)	18.00 (0.0, 33.0)	16.00 (0.0, 33.0)
**GS**	*n* = 59	*n* = 60	*n* = 57	*n* = 117
**Mean (SD)**	4.6 (1.2)	4.0 (1.8)	4.3 (1.4)	4.2 (1.6)
**Median (min, max)**	5.2 (0.0, 5.4)	5.1 (0.0, 5.4)	5.2 (0.0, 5.4)	5.2 (0.0, 5.4)
**Stool frequency**	*n* = 60	*n* = 60	*n* = 57	*n* = 117
**Mean (SD)**	2.4 (0.7)	2.3 (0.8)	2.5 (0.6)	2.4 (0.8)
**Median (min, max)**	3.0 (1.0, 3.0)	2.5 (0.0, 3.0)	3.0 (1.0, 3.0)	3.0 (0.0, 3.0)
**Rectal bleeding**	*n* = 60	*n* = 60	*n* = 57	*n* = 117
**Mean (SD)**	1.5 (0.7)	1.5 (0.8)	1.2 (0.8)	1.4 (0.8)
**Median (min, max)**	2.0 (0.0, 3.0)	2.0 (0.0, 3.0)	1.0 (0.0, 3.0)	1.0 (0.0, 3.0)
**MES**	*n* = 60	*n* = 60	*n* = 57	*n* = 117
**Mean (SD)**	2.7 (0.4)	2.7 (0.5)	2.7 (0.4)	2.7 (0.5)
**Median (min, max)**	3.0 (2.0, 3.0)	3.0 (2.0, 3.0)	3.0 (2.0, 3.0)	3.0 (2.0, 3.0)
**UCEIS**	*n* = 60	*n* = 60	*n* = 57	*n* = 117
**Mean (SD)**	5.0 (1.1)	4.5 (1.0)	4.9 (1.2)	4.7 (1.1)
**Median (min, max)**	5.0 (3.0, 8.0)	5.0 (2.0, 6.0)	5.0 (2.0, 8.0)	5.0 (2.0, 8.0)
**NHI**	*n* = 59	*n* = 60	*n* = 57	*n* = 117
**Mean (SD)**	3.2 (0.9)	2.8 (1.3)	3.0 (1.0)	2.9 (1.2)
**Median (min, max)**	3.0 (0.0, 4.0)	3.0 (0.0, 4.0)	3.0 (0.0, 4.0)	3.0 (0.0, 4.0)
**UC-100**	*n* = 59	*n* = 60	*n* = 56	*n* = 116
**Mean (SD)**	73.7 (17.0)	69.0 (18.2)	75.2 (14.2)	72.0 (16.6)
**Median (min, max)**	78.0 (29.0, 100.0)	70.0 (29.0, 100.0)	77.5 (37.0, 100.0)	72.0 (29.0, 100.0)

Abbreviations: CRP, C-reactive protein; FCAL, fecal calprotectin; GS, Geboes Score; max, maximum; MES, Mayo Endoscopic Subscore; min, minimum; NHI, Nancy Histological Index; RHI, Robarts Histopathology Index; SD, standard deviation; UCEIS, Ulcerative Colitis Endoscopic Index of Severity.

### Responsiveness

Data on the responsiveness of UC disease activity indices, expressed as WinP estimates, are summarized in Table 2. For endoscopic index responsiveness assessed using the pooled 200 mg and 600 mg mirikizumab treatment arms, the UCEIS demonstrated a “medium” degree of responsiveness (WinP estimate 0.67; 95% CI 0.59, 0.74), whereas the MES demonstrated a “small” degree of responsiveness (WinP estimate 0.61; 95% CI 0.53, 0.68). The difference in WinP estimates between the UCEIS and MES was statistically significant (0.06; 95% CI 0.02, 0.11; *P *= 0.009), suggesting that the UCEIS was more responsive than the MES (Table 3). Among histologic indices, the GS (WinP estimate 0.60; 95% CI 0.52, 0.68) and the RHI (WinP estimate 0.62; 95% CI 0.54, 0.70) demonstrated “small” responsiveness, although the responsiveness of the NHI (WinP estimate 0.57) was not better than chance alone, with a 95% CI (0.48, 0.65) containing 0.50 (Table 2). There were no statistically significant differences in responsiveness among the RHI and GS (*P *= 0.07) or among the RHI and NHI (*P *= 0.07) (Table 3).

**Table 2:** Responsiveness of UC indices for discriminating treatment with mirikizumab (pooled 200 and 600 mg doses) from placebo at week 12.

Index	WinP^a^	Lower limit for 95% CI WinP	Upper limit for 95% CI WinP	*P*-Value for WinP = 0.5
**MES**	0.61	0.53	0.68	0.006
**UCEIS**	0.67	0.59	0.74	< 0.001
**GS**	0.60	0.52	0.68	0.02
**RHI**	0.62	0.54	0.70	0.005
**NHI**	0.57	0.48	0.65	0.12
**UC-100**	0.68	0.60	0.75	< 0.001

Abbreviations: CI, confidence interval; GS, Geboes Score; MES, Mayo Endoscopic Subscore; NHI, Nancy Histological Index; RHI, Robarts Histopathology Index; UC, ulcerative colitis; UCEIS, Ulcerative Colitis Endoscopic Index of Severity; WinP, win probability.

a WinP estimates of 0.50, 0.56, 0.64, and 0.71 were interpreted as “null,” “small,” “medium,” and “large” effect sizes, respectively.

**Table 3:** Comparison of responsiveness between UC indices for discriminating treatment with mirikizumab (pooled 200 and 600 mg doses) from placebo, estimated by differences in win probability.

Comparison of indices	Difference in WinP	Lower limit for 95% CI difference	Upper limit for 95% CI difference	*P*-Value for WinP1 = WinP2
**UCEIS vs MES**	0.06	0.02	0.11	0.009
**RHI vs GS**	0.02	0	0.05	0.07
**RHI vs NHI**	0.06	0	0.12	0.07
**UC-100 vs MES**	0.07	0	0.15	0.05
**UC-100 vs UCEIS**	0.01	−0.06	0.08	0.73
**UC-100 vs GS**	0.08	0.02	0.14	0.008
**UC-100 vs RHI**	0.06	0.01	0.11	0.03
**UC-100 vs NHI**	0.12	0.04	0.19	0.003

Abbreviations: CI, confidence interval; GS, Geboes Score; MES, Mayo Endoscopic Subscore; NHI, Nancy Histological Index; RHI, Robarts Histopathology Index; UC, ulcerative colitis; UCEIS, Ulcerative Colitis Endoscopic Index of Severity; WinP, win probability.

The composite UC-100 score demonstrated “medium” responsiveness (WinP estimate 0.68; 95% CI 0.60, 0.75) (Table 2). Statistically significant differences in responsiveness were observed between the UC-100 score and the histologic indices (RHI, GS, and NHI) but not between the UC-100 score and the endoscopic indices (UCEIS and MES) (Table 3).

### Longitudinal validity

On pooled analysis (combined data from the mirikizumab and placebo arms), the MES, UCEIS, RHI, GS, and UC-100 score had statistically significant correlations with clinical symptoms (SF and/or RB) (Table 4). However, the NHI displayed a significant correlation with SF only (0.23; 95% CI 0.08, 0.36) and not with RB (0.13; 95% CI −0.02, 0.27). The UC-100 score showed a moderate correlation with RB (0.46; 95% CI 0.34, 0.57), while the UCEIS demonstrated a moderate correlation with RB (0.33; 95% CI 0.19, 0.46). The remaining correlations were weak in magnitude.

**Table 4:** Spearman rank correlation coefficients (95% CIs) between the change in index score and changes in clinical and biomarker outcomes.

Index	Treatment assignment	SF	RB	FCAL	CRP
**MES**	Overall^a^	0.21 (0.06, 0.34)	0.29 (0.15, 0.42)	0 (−0.22, 0.23)	0.02 (−0.13, 0.17)
	Pooled mirikizumab^b^	0.18 (0, 0.35)	0.25 (0.07, 0.41)	−0.05 (−0.32, 0.23)	−0.16 (−0.33, 0.03)
**UCEIS**	Overall^a^	0.26 (0.11, 0.39)	0.33 (0.19, 0.46)	0.17 (−0.06, 0.38)	0.14 (−0.01, 0.28)
	Pooled mirikizumab^b^	0.23 (0.05, 0.40)	0.28 (0.11, 0.44)	0.06 (−0.23, 0.33)	0.06 (−0.13, 0.24)
**GS**	Overall^a^	0.27 (0.12, 0.40)	0.19 (0.04, 0.33)	0.23 (0.01, 0.43)	0.05 (−0.10, 0.20)
	Pooled mirikizumab^b^	0.24 (0.06, 0.40)	0.17 (−0.01, 0.34)	0.19 (−0.09, 0.44)	−0.07 (−0.25, 0.11)
**RHI**	Overall^a^	0.22 (0.08, 0.36)	0.19 (0.04, 0.33)	0.23 (0.01, 0.43)	0.08 (−0.08, 0.22)
	Pooled mirikizumab^b^	0.23 (0.05, 0.40)	0.21 (0.03, 0.38)	0.29 (0, 0.52)	−0.03 (−0.22, 0.15)
**NHI**	Overall^a^	0.23 (0.08, 0.36)	0.13 (−0.02, 0.27)	0.17 (−0.05, 0.38)	0.08 (−0.07, 0.23)
	Pooled mirikizumab^b^	0.24 (0.06, 0.40)	0.17 (−0.02, 0.34)	0.05 (−0.24, 0.32)	0 (−0.18, 0.18)
**UC-100**	Overall^a^	–	0.46 (0.34, 0.57)	0.42 (0.21, 0.58)	0.19 (0.05, 0.33)
	Pooled mirikizumab^b^	–	0.42 (0.26, 0.56)	0.39 (0.11, 0.60)	0.04 (−0.14, 0.23)

Abbreviations: CRP, C-reactive protein; FCAL, fecal calprotectin; GS, Geboes Score; MES, Mayo Endoscopic Subscore; NHI, Nancy Histological Index; RB, rectal bleeding; RHI, Robarts Histopathology Index; SF, stool frequency; UC, ulcerative colitis; UCEIS, Ulcerative Colitis Endoscopic Index of Severity.

a Spearman correlation using data from pooled mirikizumab arms (200 mg and 600 mg) and placebo arm.

b Spearman correlation using data from pooled mirikizumab arms (200 mg and 600 mg).

For biomarker outcomes, neither the MES nor the UCEIS showed a significant correlation with CRP or FCAL (Table 4). Among the histologic indices, the GS and RHI demonstrated weak and significant correlations with FCAL level, while the NHI did not show statistically significant correlations with biomarker outcomes. Additionally, none of the histologic indices correlated with CRP level. However, the UC-100 score demonstrated a weak correlation with CRP level and moderate correlation with FCAL level.

## Discussion

Despite the availability of several biologics and oral small molecules, evidence suggests that a therapeutic ceiling in the treatment of UC has been reached, with one-year remission rates generally not surpassing 50% in phase 3 clinical trials.^22^ Therefore, in an attempt to address this unmet need, there is continuing development of therapies and treatment strategies with the goal of breaking this therapeutic ceiling. Unfortunately, only approximately 25% of drugs successfully transition from the earliest phases of drug development to reach phase 3 testing,^23^ and the decisions required for phase transition rely on the availability of highly responsive, well-validated assessment tools that can facilitate go-no/go decisions. Despite the development of approximately 20 endoscopic disease activity assessment indices for UC, none have been fully validated.^24^ The MES remains the most commonly used instrument in clinical trials for the assessment of endoscopic disease activity. Mucosal healing has been recognized by researchers and regulatory authorities as an important endpoint in both clinical trials and in the context of improved long-term outcomes. In the present study, we found that the MES demonstrated only small responsiveness to treatment-induced changes, whereas the more recently developed UCEIS index displayed measurably better responsiveness. Our data also indicate that histologic indices demonstrated only small responsiveness, whereas the composite UC-100 score, comprising the SF, RHI, and MES, demonstrated greater responsiveness, consistent with previous observations.^25^

The operating characteristics of endoscopic indices should be clearly demonstrated to inform clinical trial design. The MES was developed on an ordinal scale, unlike the UCEIS, which has a wider range of scores (from 0 to 8) and was developed using rigorous methodology.^5^ Reliability studies have demonstrated moderate to substantial inter- and intra-rater reliability of the MES, and one study that assessed intra-rater reliability indicated improved reliability with training.^26^^,^^27^ The UCEIS was developed using a linear mixed regression model which assessed the extent of endoscopic severity using three variables that account for 90% of the overall assessment of endoscopic severity judged by a VAS. The UCEIS has been demonstrated to display good intra-rater agreement (κ  =  0.72) and moderate inter-rater agreement (κ  =  0.50).^6^ However, very few studies have assessed the responsiveness of the modified MES and the UCEIS.^28^^,^^29^ In a study by Levesque et al., the MES and the UCEIS displayed similar small to moderate responsiveness based on Cohen’s effect size, Guyatt’s responsiveness statistic for the assessment of UC disease activity, despite the UCEIS having greater numerical value.^28^ In the present study, the UCEIS demonstrated better responsiveness than the MES. This finding may reflect the wider range of UCEIS scores and more granular measurements of endoscopic disease activity relative to the simpler MES, although the UCEIS has practical considerations such as increased scoring complexity. The UCEIS also demonstrated a greater ability to differentiate between the treatment and placebo arms in the context of endoscopic index improvement. Considering anticipated advancements, while AI-assisted endoscopic interpretation and multimodal modeling are expected to transform assessment in UC, validated scoring systems such as the UCEIS will remain essential for regulatory alignment and trial comparability and to help develop and calibrate future AI models.

Histologic disease activity persists in approximately 25% of patients who have achieved endoscopic mucosal healing.^9^^,^^12^ Several observational studies demonstrated that achievement of histologic remission was associated with improved long-term outcomes, whereas persistent histologic inflammation was associated with corticosteroid use, hospitalization, and the development of colorectal cancer.^8^^,^^30^^,^^31^ However, histologic improvement of disease activity is not currently considered as a treatment target and is not mandated by regulatory authorities as an endpoint in clinical trials.^2^ Despite the availability of validated instruments, variations in sampling and in the methods used to procure biopsies may result in greater variability with histologic endpoints than with endoscopy. Moreover, the feasibility and therapeutic benefits of achieving histologic remission are yet to be demonstrated. The continuing VERDICT trial (NCT04259138) has the potential to determine the optimal treatment target in UC, with reference to symptomatic, endoscopic, and histologic remission.^32^ In a post hoc analysis from a phase 2 study assessing the efficacy of ozanimod (TOUCHSTONE), histologic indices including the RHI, GS, NHI, and modified Riley score were found to display modest responsiveness, and the effect sizes improved with changing definitions of response.^33^ In the present study, the GS and RHI displayed small responsiveness, and the NHI did not demonstrate statistically significant responsiveness. Potential reasons for these discrepancies include the use of a single data set in our analysis with a small sample size or the possibility that histologic change may be more difficult or take longer to attain, especially during the induction phase. Indeed, histology may have greater utility at later maintenance timepoints, whereas clinical and endoscopic components may drive responsiveness during induction.

The UC-100 score was developed and validated as a composite disease activity index with good discriminative performance, with the goal of using this tool in early phase trials of UC.^14^ This index features a continuous scale with a score ranging from 1 (no disease activity) to 100 (severe disease activity). We found the UC-100 score to be the most numerically responsive instrument assessed in this study, with medium responsiveness that correlated with clinical symptoms and biomarker levels. The combination of clinical, endoscopic, and histologic items in the UC-100 score may provide additive value and has previously been proposed as an improved measure of disease activity relative to the individual application of these items.^14^ A post hoc analysis of a large sample (*n* = 961) demonstrated a larger degree of responsiveness (WinP 0.72; 95% CI 0.66, 0.78) for the UC-100 score than for the MCS, indicating that the UC-100 score may be more appropriate for measuring treatment effects when an objective continuous measure is required.^25^

Our study has several strengths. First, the data used for these analyses were derived from a well-conducted, phase 2, randomized controlled trial of a highly effective therapy that included centrally evaluated histopathology and endoscopy, and with collected and measured patient-reported outcomes. Second, we have used appropriate statistical methods for quantifying instrument responsiveness. However, we also acknowledge some important limitations of this study. This was a post hoc analysis, and the original study was not specifically powered for comparing treatment responsiveness of different disease activity indices. Due to the relatively small sample size, our analyses may be underpowered to detect small differences in responsiveness between different instruments. We observed poor correlations between the disease activity indices and biomarker levels, and these findings could be related to inter- and intra-individual variability in biomarker measurements.^34^

In conclusion, the UCEIS and UC-100 score were responsive instruments in patients with UC for measuring changes in endoscopic and global disease activity, respectively. The UCEIS demonstrated better responsiveness than the traditional MES score and a greater ability to differentiate between treatment and placebo arms in the context of endoscopic index improvement in this phase 2 trial. The composite UC-100 score may be used as a secondary endpoint in randomized controlled trials or as a potential primary outcome in early phase trials. Future studies with a larger sample size should be considered to confirm findings of this study. We anticipate that these results will be helpful in informing the choice of measurement tools in phase 2 clinical trials of UC and in the design and conduct of more efficient trials.

## Supplementary Material

otag031_Supplementary_Data

## Data Availability

All data for this post hoc analysis were made available by Eli Lilly and Company. All analyses, interpretation, and manuscript drafting were performed independently by the study authors. The data supporting the findings of this study must be requested from Eli Lilly and Company.

## Writing assistance

Medical writing support was provided by Claire E. Parker and Stefanie C. McFarlane of Alimentiv Inc. and was funded by Alimentiv Inc.
