# Supplementary material for: The UCEIS and UC-100 score were responsive endoscopic and global indices in a phase 2 trial of ulcerative colitis
Source: Crohns Colitis 360. 2026 Apr 24;8(2):otag031. doi: 10.1093/crocol/otag031 (PMC13197579; doi:10.1093/crocol/otag031)
Supplement: otag031_Supplementary_Data [file otag031_supplementary_data.zip › RCT01464-SUPPLEMENT-18JUN2025.docx]

# SUPPLEMENTARY MATERIAL

## Supplementary Table 1. Endoscopic, histologic, and composite indices

| **Index** | **Item** | **Scale** | **Scoring** |
| --- | --- | --- | --- |
| ***Endoscopic indices*** | | | |
| MES | **Total** | 0-3 | 0, normal or inactive disease; 1, mild disease (erythema, decreased vascular pattern, no friability); 2, moderate disease (marked erythema, absent vascular pattern, friability, erosions); 3, severe (spontaneous bleeding, ulceration) |
| UCEIS | **Total** | 0-8 | Sum of item scores |
|  | Vascular pattern | 0-2 | 0, normal (normal vascular pattern with arborisation of capillaries clearly defined or with blurring or patchy loss of capillary margins); 1, patchy obliteration (patchy obliteration of vascular pattern); 2, obliterated (complete obliteration of vascular pattern) |
|  | Bleeding | 0-3 | 0, none (no visible blood); 1, mucosal (some spots or streaks of coagulated blood on the surface of the mucosa ahead of the scope that can be washed away; 2, luminal mild (some free liquid blood in the lumen); 3, luminal moderate or severe (frank blood in the lumen ahead of endoscope or visible oozing from mucosa after washing intraluminal blood, or visible oozing from a haemorrhagic mucosa) |
|  | Erosions and ulcers | 0-3 | 0, none (normal mucosa, no visible erosions or ulcers); 1, erosions (tiny [≤ 5 mm] defects in the mucosa of a white or yellow colour with a flat edge); 2, superficial ulcer (larger [> 5 mm] defects in the mucosa, which are discrete fibrin-covered ulcers when compared with erosions but remain superficial); 3, deep ulcer (deeper excavated defects in the mucosa with a slightly raised edge) |
| ***Histologic indices*** | | | |
| GS | **Total** | 0.0-5.4 | Highest available valid subgrade |
|  | Grade 0: Structural (architectural change) | 0.0-0.3 | 0.0, no abnormality; 0.1, mild abnormality; 0.2, mild or moderate diffuse or multifocal abnormalities; 0.3, severe diffuse or multifocal abnormalities |
|  | Grade 1: Chronic inflammatory infiltrate | 1.0-1.3 | 1.0, no increase; 1.1, mild but unequivocal increase; 1.2, moderate increase; 1.3, marked increase |
|  | Grade 2A: Lamina propria eosinophils | 2A.0-2A.3 | 2A.0, no increase; 2A.1, mild but unequivocal increase; 2A.2, moderate increase; 2A.3, marked increase |
|  | Grade 2B: Lamina propria neutrophils | 2B.0-2B.3 | 2B.0, no increase; 2B.1, mild but unequivocal increase; 2B.2, moderate increase; 2B.3, marked increase |
|  | Grade 3: Neutrophils in epithelium | 3.0-3.3 | 3.0, none; 3.1, < 5% crypts involved; 3.2, < 50% crypts involved; 3.3, > 50% crypts involved |
|  | Grade 4: Crypt destruction | 4.0-4.3 | 4.0, none; 4.1, probable – local excess of neutrophils in part of the crypt; 4.2, probable – marked attenuation; 4.3, unequivocal crypt destruction |
|  | Grade 5: Erosion or ulceration | 5.0-5.4 | 5.0, no erosion, ulceration, or granulation tissue; 5.1, recovering epithelium + adjacent inflammation; 5.2, probable erosion – focally stripped; 5.3, unequivocal erosion; 5.4, ulcer or granulation tissue |
| NHI | **Grade** | 0-4 | 0, no histologically significant disease; 1, chronic inflammatory infiltrate with no acute inflammatory infiltrate;  2, acute inflammatory infiltrate – mildly active disease; 3, acute inflammatory infiltrate – moderately active disease;  4, ulceration – severely active disease |
|  | Ulceration (defined as visible epithelial injury and regeneration and/or fibrin and neutrophils and/or tissue granulation) | 0-1 | 0, no; 1, yes |
|  | Acute inflammatory cells infiltrate | 0-3 | 0, none; 1, mild; 2, moderate; 3, severe |
|  | Chronic inflammatory infiltrate (defined as the quantity of lymphocytes and plasmacytes in the biopsy specimen) | 0-3 | 0, no increase; 1, mild (but unequivocal) increase; 2, moderate increase; 3, marked increase |
| RHI | **Total** | 0-33 | Sum of item scores (after adjustment using multiplication factors of 1, 2, 3, and 5 for chronic inflammatory infiltrate, lamina propria neutrophils, neutrophils in epithelium, and erosion or ulceration, respectively) |
|  | Chronic inflammatory infiltrate | 0-3 | 0, no increase; 1, mild but unequivocal increase; 2, moderate increase; 3, marked increase |
|  | Lamina propria neutrophils | 0-3 | 0, none; 1, mild but unequivocal increase; 2, moderate increase; 3, marked increase |
|  | Neutrophils in epithelium | 0-3 | 0, none; 1, < 5% crypts involved; 2, < 50% crypts involved; 3, > 50% crypts involved |
|  | Erosion or ulceration | 0-3 | 0, no erosion, ulceration, or granulation tissue; 1, recovering epithelium + adjacent inflammation; 1, probable erosion – focally stripped; 2, unequivocal erosion; 3, ulcer or granulation tissue |
| ***Composite index*** | | | |
| UC-100 score | **Total** | 1-100 | 1 + (16 × Mayo SF subscore) + (6 × MES) + (1 × RHI) |
|  | Stool frequency | 0-3 | 0, patient reports a normal number of daily stools; 1, 1-2 more stools than normal; 2, 3-4 more stools than normal;  3, ≥ 5 more stools than normal |
|  | MES | 0-3 | 0, normal or inactive disease; 1, mild disease (erythema, decreased vascular pattern, no friability); 2, moderate disease (marked erythema, absent vascular pattern, friability, erosions); 3, severe (spontaneous bleeding, ulceration) |
|  | RHI | 0-33 | See RHI index scoring above |
| Abbreviations: GS, Geboes score; MES, Mayo Endoscopic Subscore; NHI, Nancy Histological Index; RHI, Robarts Histopathology Index; SF, stool frequency; UCEIS, Ulcerative Colitis Endoscopic Index of Severity. | | | |
